# Investigating Poor Sleep Quality and Associated Factors During the COVID-19 Pandemic: A Population-Based Survey in Bangladesh

**DOI:** 10.3389/fpubh.2021.724520

**Published:** 2021-11-22

**Authors:** Md. Saiful Islam, Md. Estiar Rahman, Abdullah Al Zubayer, Md. Rifat Al Mazid Bhuiyan, Md. Kamrul Ahsan Khan, Liakat Hossain, Md. Monjurul Ahasan Sujon

**Affiliations:** ^1^Department of Public Health and Informatics, Jahangirnagar University, Dhaka, Bangladesh; ^2^Centre for Advanced Research Excellence in Public Health, Dhaka, Bangladesh; ^3^Department of Sociology, University of Barishal, Barishal, Bangladesh; ^4^Dhaka Community Medical College, Dhaka, Bangladesh; ^5^Sheikh Sayera Khatun Medical College, Gopalgonj, Bangladesh; ^6^Faculty of Medicine, Dhaka Medical College and Hospital, Dhaka, Bangladesh

**Keywords:** COVID-19, sleep quality, risk factors, mental health, Bangladeshi people

## Abstract

**Background:** The coronavirus disease 2019 (COVID-19) pandemic has adversely affected the sleep quality of individuals, and is a poorly investigated area. This study aimed to investigate the prevalence estimate of poor sleep quality and its associated factors among Bangladeshi residents during the COVID-19 pandemic.

**Methods:** An online cross-sectional survey was carried out from July 20 to August 5, 2020, involving 975 Bangladeshi residents (male: 54.2%; mean age: 26.7 ± 9.4 years; age range: 18–75 years). A self-reported questionnaire was answered by the respondents, covering information on demographic characteristics, perceived physical health status, COVID-19-related factors, COVID-19-induced anxiety assessment, and sleep quality. To assess sleep quality, the Bangla version of the Pittsburgh Sleep Quality Index was used. Logistic regression models were performed to analyze the factors associated with sleep quality.

**Results:** The prevalence estimate of poor sleep quality was 55.1% among the Bangladeshi people during the COVID-19 pandemic. As per the multiple regression analysis, poor sleep quality was significantly higher among respondents who reported female gender, moderate/poor health status, indirect contact with COVID-19 infected patients, decreased household income due to the COVID-19 pandemic, fear of infection, and COVID-19-induced anxiety.

**Conclusion:** Poor sleep quality was slightly prevalent among general people in Bangladesh during the COVID-19 pandemic. The findings indicate an immediate response for this vulnerable group to improve the sleep quality during the public health emergency of COVID-19.

## Introduction

The coronavirus disease 2019 (COVID-19) outbreak, discovered in late December 2019 in Wuhan, China, has become a global public health concern. On March 11, 2020, the WHO announced COVID-19 as a pandemic (1). The virus continues to spread worldwide with more than 188 million confirmed cases and more than four million deaths as of July 16, 2021 (2).

In Bangladesh (where this study was conducted), the first case of COVID-19 was confirmed on March 8, 2020 (3, 4). To date (as of July 16, 2021), the country has reported more than 1 million confirmed cases of COVID*-*19 and 17,278 COVID-19-related deaths domestically (5). The government of Bangladesh imposed a countrywide lockdown on March 26, 2021 in order to limit the spread of COVID-19 and ended partially on May 30, 2020 (6, 7). The government has restricted all gathering activities and suggested to wear masks in public places to prevent transmission (8). Evidence shows that anxiety, depression, insomnia, stress, panic attack, and post-traumatic stress disorder have all been linked to pandemic issues such as spatial distancing, isolation, quarantine, and social and economic impacts (9–13), which can also play a dynamic role in sleep quality (14). As the pandemic progresses, pandemic-related restrictions, a rise in the number of new cases, and fear of infection are causing mental health problems in the general population (15, 16), potentially affecting sleep quality.

Sleep is a naturally occurring condition of the body and mind. A good quality of sleep is essential for sustaining good health and for strengthening the immune system (17). Conversely, inadequate sleep increases the risk of obesity, cardiovascular and metabolic conditions, and mood and cognitive problems (17–20). Inadequate sleep is considered a public health problem worldwide, being attributed to 7 of the 15 leading causes of death in the United States (21). The ongoing COVID-19 pandemic has had a significant impact on the lives of people across the world, including the way people sleep. Sleep problems were also common among both healthcare professionals (36.0%; 95% CI = 21.1–54.2%) and general people (32.3%; 95% CI = 25.3–40.2%) during the COVID-19 pandemic (22). Several studies demonstrated poor sleep quality during the COVID-19 pandemic among the general population (18.2%) in China (23) and in Italy (57.1%) (24). Likewise, elevated poor sleep quality was also found among university students (73.3%) and administration staff (60.2%) in Italy (25), university students in Jordan (76.0%) (14), and healthcare workers (75.2%) in Bahrain (26). Moreover, a global study covering 49 countries recorded 58% of those surveyed with insufficient sleep and 40% of those surveyed with decreased sleep quality during the COVID-19 pandemic. A meta-analysis of 44 studies with a total of 54,231 respondents from 13 countries found that the global pooled prevalence rate of sleep problems for all populations was 35.7% (95% CI = 29.4–42.4%) during the COVID-19 pandemic (22). According to this report, patients with COVID-19 tended to be the most affected group with a pooled rate of 74.8% (95% CI = 28.7–95.6%) (22). Approximately, 4 in every 10 people were reported to have sleep problems during the COVID-19 pandemic (22). The COVID-19 pandemic has not only exacerbated extreme poverty in lower- and middle-income countries (LMICs), but has also triggered mental health problems (27). So, it is very important to investigate sleep quality during the COVID-19 pandemic among general people in LMICs including Bangladesh, as they are dealing with economic instabilities, poverty, joblessness, food insecurity, and inability to access medicine (28–30).

During the COVID-19 pandemic, various studies conducted with different demographics in Bangladesh reported mental health problems. For instance, anxiety, depression, panic, worry, stress, suicidal ideation, and behavioral problems (such as problematic use of smartphones, internet, and social media) were prevalent in Bangladesh (11, 12, 15, 31–39). However, the sleep quality of the general population has received comparatively little attention in studies during the pre-COVID-19 period and amidst the COVID-19 pandemic. A pre-COVID-19 study recorded that the prevalence rates of poor sleep quality were 42.6 and 35.9%, respectively, among urban and rural adult populations in India using the Pittsburgh Sleep Quality Index (PSQI) (40). In Bangladesh, pre-COVID-19 studies using a similar methodology found 66.6% poor sleep quality among university students (41) and 69.5% poor sleep quality among medical students (42). Evidence suggests that the poor sleep quality was associated with female gender, urban residence, moderate/poor self-reported health status, poor quality of life, having less sleep a night, problematic internet use, and more social media use (40–44).

To the best of our knowledge, there was no study examining sleep quality during the COVID-19 pandemic in Bangladesh at the time of this study. Consequently, this study aimed to investigate the prevalence of poor sleep quality by using the PSQI and its associated factors among Bangladeshi people during the COVID-19 pandemic. However, some studies on sleep disturbance, subjective sleep quality, and insomnia have recently been published, conducted with different groups in Bangladesh (45–48). Though one of these studies assessed the sleep quality by using the PSQI among Bangladeshi general people with limited samples, it did not investigate any COVID-19 pandemic-related factors and their relations with poor sleep quality (48). We would hypothesize that poor sleep quality would be higher among females, urban residents, those with moderate/poor health status, and associated with other sociodemographic factors (e.g., age, education, marital status, occupation, etc.). It was also hypothesized that there would be positive correlations between poor sleep quality and COVID-19-related factors (e.g., economic impacts due to the COVID-19, fear of infection, COVID-19 anxiety, etc.).

## Materials and Methods

### Study Design and Population

This was an online cross-sectional survey that investigated the sleep quality among 975 Bangladeshi residents during the COVID-19 pandemic. The survey was conducted from July 20 to August 5, 2020, targeting the individuals who resided in Bangladesh. The inclusion criteria was as follows: (i) being a Bangladeshi resident, (ii) being an adult (≥18 years), (iii) having the ability to read Bangla (as the survey was written in Bangla language), and (iv) being willing to take part in the survey. The exclusion criteria were being under 18 years and not completing the questionnaire entirely.

### Sampling

The sample size was calculated using the RaoSoft® (RaoSoft, Inc., Seattle, WA), an online sample size calculator (49, 50). As there was no prior similar study at the time of the study focusing on individuals who resided in Bangladesh, we estimated that half of the subjects (50%) would have poor sleep quality. The minimum required sample size for this study was 384 with 95% confidence level and a margin of error of 5%. However, we finally recruited 975 samples using the convenience sampling technique.

### Survey Procedures

This online survey was conducted using the Google survey tool (Google Forms). Respondents were recruited from different social media sites (e.g., Facebook, Messenger, WhatsApp, etc.). Data were collected utilizing an anonymous and self-reported e-questionnaire writing in Bangla (first language of the respondents). A sample of 40 respondents was piloted for the survey to test the validity of the questionnaire. Following the pilot test, some minor modifications (e.g., spelling corrections) were incorporated in the survey questionnaire based on the feedback of the participants. These surveys from the pilot test were excluded in the final analysis. Initially, a total of 1,070 respondents took part in the survey without any financial compensation. After removing incomplete and data-missing surveys, 975 respondents [54.2% male; mean age: 26.7 years (SD = 9.4); age range: 18–75 years] were included in the final analysis.

### Ethical Considerations

This study was carried out in accordance with the Helsinki Declaration and Institutional Research Ethics guidelines. In addition, the study protocol was reviewed and approved by the Institutional Review Board of Sheikh Sayera Khatun Medical College, Gopalganj, Bangladesh [SSKMC/EC/2020/810]. All the respondents were informed about the aims and objectives of study and e-informed consent was obtained from everyone prior to the survey.

### Measures

#### Background Variables

Background variables were inquired through both the open- and close-ended questions. Respondents were asked to report their age, gender (male vs. female), relationship status (married vs. unmarried), education (college or bellow/university or higher), occupation (student/housewife/employee/health workers/businessman/unemployed), family type (nuclear vs. joint/extended), socio-economic status (SES) (categorized based on monthly family income: lower SES <15,000 Bangladeshi Taka [BDT], middle SES = 15,000-30,000 BDT, and upper SES > 30,000 BDT) (51, 52), and residence (rural vs. urban). Other variables included: perceived physical health status (good/moderate/poor), tobacco smoking (yes/no), and alcohol consumption (yes/no).

#### Experiences of the Respondents Amidst the COVID-19 Pandemic

With regard to the personal experiences of the respondents due to the COVID-19 pandemic, “yes/no” questions were asked during the survey, including: (i) did any relatives or acquaintances get infected with COVID-19, (ii) did any relatives or acquaintances die from COVID-19 infection, (iii) was there any contact with patients with COVID-19 directly (16), (iv) was there any contact indirectly with patients with COVID-19 (16), and (v) were there any household income decreases due to COVID-19. In addition, another “yes/no” question was asked to evaluate the respondents' fear of COVID-19 infection (*i.e., are you afraid that you could be infected with COVID-19?*).

#### Coronavirus Anxiety Scale (CAS)

The CAS is a unidimensional psychometric screening tool for assessing dysfunctional anxiety resulting from the current COVID-19 pandemic (53). This scale consists of five-item questions concerning problems related to anxiety symptomatology due to COVID-19 over the past 2 weeks (e.g., “*I lost interest in eating when I thought about or was exposed to information about the coronavirus*”) with a five-point Likert scale ranging from 0 (“*not at all*”) to 4 (“*nearly every day over the last 2 weeks*”). The cutoff ≥ 9 demonstrated very satisfactory sensitivity (90%) and specificity (85%) (53). In this study, the validated Bangla version of the CAS was used to assess the COVID-19-induced anxiety of the respondents (54). The Cronbach's alpha of the CAS was 0.82, indicating a good internal consistency.

#### Pittsburgh Sleep Quality Index (PSQI)

The PSQI is a widely used self-reported instrument for assessing sleep quality (55). It comprises 19 items questions including seven components (i.e., subjective sleep quality, sleep latency, sleep duration, habitual sleep efficiency, use of sleep medication, and daytime dysfunction), which are related to the features of sleep quality over the last month (55), each weighted equally on a 0–3 scale. The global PSQI score of sleep quality is yielded by summating the scores of the seven components ranging from 0 to 21. A higher score indicates poor sleep quality and a lower score reflects good sleep quality (55). In this study, the validated Bangla version of the PSQI was used to assess sleep quality (40) as previously in Bangladesh (42, 44). The PSQI score > 5 was used as the cutoff for the poor sleep quality (40, 44).

### Statistical Analysis

Statistical analysis was carried out by using the Statistical Package for the Social Sciences (SPSS) IBM Statistics version 25.0 (Armonk, NY, USA). Means and SDs were calculated for continuous variables; in contrast, frequencies and percentages were calculated for categorical variables. For categorical comparisons of variables, the chi-squared test was executed. In addition, the multiple logistic regression analysis was conducted with a 95% CI to determine the associated factors of poor sleep quality by using three separate models (i.e., model 1, model 2, and model 3). Model 1 comprised only background variables, while only COVID-19-related variables were included in model 2. Finally, both the background and COVID-19-related variables were included in model 3. The poor sleep quality (PSQI score > 5) was used as a dependent variable for each model. The reason for these models was to investigate the combined effects of only background variables, only the COVID-19-related variables, and both the background and the COVID-19-related variables by model 1, model 2, and model 3, respectively. The two-sided *p* < 0.05 was deemed as statistically significant for all the analyses.

## Results

A total of 975 respondents were included in the final analysis. The mean age of the respondents was 26.7 years (SD = 9.4; age range = 18–75 years) and more than half of them (54.2%) were male. The descriptive statistics of all the variables are given in Table 1.

**Table 1:** Descriptive analyses for all the examined variables with sleep quality (*n* = 975).

**Variables**		**Sleep quality**						
	**Total** ***N*** **=** **975**		***Good***		***Poor***		**χ^2^**	***df***	***p*-value**
	***n***	**(%)**	***n***	**(%)**	***N***	**(%)**			
**Age**									
18–25 years	631	(64.7)	295	(46.8)	336	(53.2)	2.42	1	0.120
>25 years	344	(35.3)	143	(41.6)	201	(58.4)			
**Gender**									
Male	528	(54.2)	274	(51.9)	254	(48.1)	22.62	1	<0.001
Female	447	(45.8)	164	(36.7)	283	(63.3)			
**Marital status**									
Married	258	(26.5)	114	(44.2)	144	(55.8)	0.08	1	0.781
Unmarried	717	(73.5)	324	(45.2)	393	(54.8)			
**Education**									
College or bellow	213	(21.8)	113	(53.1)	100	(46.9)	7.28	1	0.007
University or higher	762	(78.2)	325	(42.7)	437	(57.3)			
**Occupation**									
Student	605	(62.1)	286	(47.3)	319	(52.7)	24.34	5	<0.001
Housewife	56	(5.7)	30	(53.6)	26	(46.4)			
Employee	142	(14.6)	56	(39.4)	86	(60.6)			
Health workers	103	(10.6)	28	(27.2)	75	(72.8)			
Businessman	44	(4.5)	28	(63.6)	16	(36.4)			
Unemployed	25	(2.6)	10	(40.0)	15	(60.0)			
**Family type**									
Nuclear	716	(73.4)	309	(43.2)	407	(56.8)	3.40	1	0.065
Joint	259	(26.6)	129	(49.8)	130	(50.2)			
**Socioeconomic status (SES)**									
Lower SES	130	(13.3)	54	(41.5)	76	(58.5)	4.56	2	0.102
Middle SES	236	(24.2)	120	(50.8)	116	(49.2)			
Upper SES	609	(62.5)	264	(43.3)	345	(56.7)			
**Residence**									
Rural	299	(30.7)	167	(55.9)	132	(44.1)	20.82	1	<0.001
Urban	676	(69.3)	271	(40.1)	405	(59.9)			
**Health status**									
Good	608	(62.4)	324	(53.3)	284	(46.7)	49.27	2	<0.001
Moderate	343	(35.2)	111	(32.4)	232	(67.6)			
Poor	24	(2.5)	3	(12.5)	21	(87.5)			
**Tobacco smoking**									
Yes	180	(18.5)	87	(48.3)	93	(51.7)	1.04	1	0.308
No	795	(81.5)	351	(44.2)	444	(55.8)			
**Alcohol consumption**									
Yes	55	(5.6)	22	(40.0)	33	(60.0)	0.57	1	0.450
No	920	(94.4)	416	(45.2)	504	(54.8)			
**Having COVID-19 infected relatives or acquaintance**									
Yes	298	(30.6)	93	(31.2)	205	(68.8)	32.63	1	<0.001
No	677	(69.4)	345	(51.0)	332	(49.0)			
**Having relatives or acquaintance died from COVID-19 infection**									
Yes	76	(7.8)	24	(31.6)	52	(68.4)	5.93	1	0.015
No	899	(92.2)	414	(46.1)	485	(53.9)			
**Direct contacts with COVID-19 patients**									
Yes	145	(14.9)	36	(24.8)	109	(75.2)	27.80	1	<0.001
No	830	(85.1)	402	(48.4)	428	(51.6)			
**Indirect contacts with COVID-19 patients**									
Yes	258	(26.5)	77	(29.8)	181	(70.2)	32.24	1	<0.001
No	717	(73.5)	361	(50.3)	356	(49.7)			
**Household income decreased due to the COVID-19 pandemic**									
Yes	723	(74.2)	309	(42.7)	414	(57.3)	5.40	1	0.020
No	252	(25.8)	129	(51.2)	123	(48.8)			
**Having fear of COVID-19**									
Yes	576	(59.1)	212	(36.8)	364	(63.2)	37.49	1	<0.001
No	399	(40.9)	226	(56.6)	173	(43.4)			
**COVID-19 induced anxiety**									
Negative	923	(94.7)	433	(46.9)	490	(53.1)	27.68	1	<0.001
Positive	52	(5.3)	5	(9.6)	47	(90.4)			

Based on the CAS, 5.3% of respondents experienced COVID-19-induced anxiety. Furthermore, a large portion (59.1%) reported that they were afraid of COVID-19 infection (Table 1). Based on the PSQI, the prevalence estimates of poor and good sleep quality were 55.1 and 44.9%, respectively. Table 1 represents the findings of bivariate analysis of the sleep quality.

The results of regression analyses were separated into three models on the basis of varying factors and exhibited several variables of interest that had statistically significant effects on poor sleep quality (see Table 2). Model 1 is adjusted for the background variables only, whereas model 2 is adjusted for the COVID-19-related variables only. Finally, model 3 is adjusted for both the background and the COVID-19-related variables.

**Table 2:** The multivariable analyses of all the variables by poor sleep quality.

**Variables**	**Model 1^a^**		**Model 2^b^**		**Model 3^c^**	
	**AOR**	**95% CI**	**AOR**	**95% CI**	**AOR**	**95% CI**
**Background variables**						
**Age**						
18–25 years	0.78	(0.48–1.25)	–	–	0.79	(0.49–1.28)
>25 years	Reference				Reference	
**Gender**						
Female	1.93^***^	(1.41–2.66)	–	–	1.78^**^	(1.28–2.48)
Male	Reference				Reference	
**Marital status**						
Married	0.78	(0.49–1.24)	–	–	0.73	(0.45–1.17)
Unmarried	Reference				Reference	
**Education**						
University or higher	1.16	(0.79–1.70)	–	–	1.12	(0.75–1.68)
College or bellow	Reference				Reference	
**Occupation**						
Housewife	0.81	(0.35–1.88)	–	–	1.04	(0.43–2.49)
Employee	1.34	(0.78–2.30)	–	–	1.39	(0.80–2.43)
Health workers	1.71	(0.97–3.01)	–	–	1.14	(0.62–2.11)
Businessman	0.54	(0.23–1.24)	–	–	0.58	(0.25–1.37)
Unemployed	1.16	(0.45–3.01)	–	–	1.46	(0.55–3.90)
Student	Reference				Reference	
**Family type**						
Nuclear	1.04	(0.76–1.42)	–	–	1.05	(0.76–1.45)
Joint	Reference				Reference	
**Socio-economic status (SES)**						
Lower SES	0.64	(0.40–1.03)	–	–	0.74	(0.45–1.21)
Middle SES	0.75	(0.48–1.19)	–	–	0.84	(0.52–1.35)
Upper SES	Reference				Reference	
**Residence**						
Urban	1.54^*^	(1.11–2.14)	–	–	1.40	(0.99–1.98)
Rural	Reference				Reference	
**Health status**						
Moderate	2.34^***^	(1.75–3.13)	–	–	2.07^***^	(1.53–2.81)
Poor	8.96^**^	(2.47–32.45)	–	–	6.76^**^	(1.73–26.37)
Good	Reference				Reference	
**Tobacco smoking**						
Yes	1.05	(0.68–1.63)	–	–	0.96	(0.60–1.51)
No	Reference				Reference	
**Alcohol consumption**						
Yes	1.53	(0.79–2.95)	–	–	1.17	(0.59–2.31)
No	Reference				Reference	
**COVID-19 related variables**						
**Having COVID-19 infected relatives or acquaintance**						
Yes	–	–	1.62^**^	(1.15–2.28)	1.41	(0.98–2.03)
No			Reference		Reference	
**Having relatives or acquaintance died from COVID-19 infection**
Yes	–	–	1.06	(0.61–1.86)	0.94	(0.52–1.68)
No			Reference		Reference	
**Direct contacts with COVID-19 patients**						
Yes	–	–	1.47	(0.88–2.46)	1.24	(0.71–2.15)
No			Reference		Reference	
**Indirect contacts with COVID-19 patients**						
Yes	–	–	1.56^*^	(1.06–2.31)	1.54^*^	(1.02–2.32)
No			Reference		Reference	
**Household income decreased due to the COVID-19 pandemic**						
Yes	–	–	1.38^*^	(1.02–1.88)	1.60^**^	(1.15–2.23)
No			Reference		Reference	
**Having fear of COVID-19**						
Yes	–	–	1.88^***^	(1.43–2.47)	1.62^**^	(1.21–2.17)
No			Reference		Reference	
**COVID-19 induced anxiety**						
Positive	–	–	6.63^***^	(2.57–17.11)	5.24^**^	(1.95–14.09)
Negative			Reference		Reference	

*AOR, adjusted odds ratio*.

* *p < 0.05*,

** *p < 0.01*,

*** *p < 0.001*.

a *Model 1 adjusted for background variables only*.

b *Model 2 adjusted for the coronavirus disease 2019 (COVID-19)-related variables only*.

c *Model 3 adjusted for both the background and the COVID-19-related variables*.

In model 1, being female [adjusted odds ratio (AOR) = 1.93; 95% CI = 1.41–2.66, *p* < 0.001], having urban residence (AOR = 1.54; 95% CI = 1.11–2.14, *p* < 0.05), and moderate and poor physical health (AOR = 2.34; 95% CI = 1.75–3.12, *p* < 0.001 and AOR = 8.96; 95% CI = 2.47–32.45, *p* < 0.01, respectively) had greater odds of poor sleep quality.

In model 2, having relatives or acquaintances who got COVID-19 infection (AOR = 1.62; 95% CI = 1.15–2.28, *p* < 0.01), having indirect contact with patients with COVID-19 (AOR = 1.56; 95% CI = 1.06–2.31, *p* < 0.05), having decreased household income due to the COVID-19 pandemic (AOR = 1.38; 95% CI = 1.02–1.88, *p* < 0.05), having fear of getting COVID-19 infection (AOR = 1.88; 95% CI = 1.43–2.47, *p* < 0.001), and having COVID-19-induced anxiety (AOR = 6.63; 95% CI = 2.57–17.11, *p* < 0.001) exhibited higher odds of poor sleep quality (Table 2).

In model 3, the effects of both the background and COVID-19-related variables were assessed. Females were 1.78 times more likely to have poor sleep quality compared to males (AOR = 1.78; 95% CI = 1.28–2.48, *p* < 0.01). Participants with moderate/poor physical health were 2.07 and 6.76 times more likely to have poor sleep quality than those had good physical health (AOR = 2.07; 95% CI = 1.53–2.81, *p* < 0.001 and AOR = 6.76; 95% CI = 1.73–26.37, *p* < 0.01, respectively). Those who had indirect contact with infected patients with COVID-19 were 1.54 times more likely to have poor sleep quality compared to those who had not had indirect contact with infected patients with COVID-19 (AOR = 1.54; 95% CI = 1.02–2.32, *p* < 0.05). Participants with decreased household income due to the COVID-19 pandemic had greater odds of poor sleep quality than those who had no decreased household income due to the COVID-19 pandemic (AOR = 1.60; 95% CI = 1.15–2.23, *p* < 0.01). Moreover, participants with fear of getting COVID-19 infection (AOR = 1.62; 95% CI = 1.21–2.17, *p* < 0.01) and COVID-19-induced anxiety (AOR = 5.24; 95% CI = 1.95–14.09, *p* < 0.01) had greater odds of poor sleep quality compared to those who had no fear of getting COVID-19 and COVID-19-induced anxiety (Table 2).

## Discussion

Globally, poor sleep quality has emerged among various groups of people, mostly in the COVID-19 pandemic (14, 23–26, 56). This study investigated the prevalence of poor sleep quality by using the PSQI among community residents in Bangladesh during the COVID-19 pandemic and its relations with sociodemographic and COVID-19 pandemic-related factors. As per present findings, 55.1% respondents experienced poor sleep quality during the COVID-19 pandemic. The multiple logistic regression analyses revealed that poor sleep quality was significantly higher among participants who reported being female, along with having moderate/poor health status, indirect contact with COVID-19-infected persons, decreased household income due to the impact of COVID-19, fear of infection, and COVID-19-induced anxiety.

The prevalence estimate of poor sleep quality (55.1%) is elevated in this study compared to the pre-COVID-19 study in India (35.9–42.6%) (40). In contrast, the prevalence estimate of this study is slightly lower than in Bangladesh among university students (55.1 vs. 66.6%) (41) along with medical students (55.1 vs. 69.5%) (42). The controversies regarding the prevalence of poor sleep quality in Bangladesh warrant a prospective study. When comparing with studies conducted during the COVID-19 pandemic in other jurisdictions, the prevalence estimate of poor sleep quality in this study is higher compared to Chinese general people (55.1 vs. 18.2%) (23) and slightly lower than Italian general people (55.1 vs. 57.1%) (24). Moreover, the present finding somewhat corresponds to a global study with 49 countries (40% decreased sleep quality) during the COVID-19 pandemic (56). In this study, the possible reason for the elevated poor sleep quality would be the frequent COVID-19 infection in Bangladesh (5).

During analysis, three separate models were performed to investigate the combined effects of only background variables, only the COVID-19-related variables, and both the background and COVID-19-related variables by model 1, model 2, and model 3, respectively. To compare the similarities and differences with previous studies, the findings from model 3 (adjusted for both the background and COVID-19-related variables) were discussed.

This study showed that females were at greater risk of poor sleep quality compared to males, which supports international studies conducted during the COVID-19 pandemic (14, 24, 26, 56) and the national pre-COVID-19 study (41). Moreover, a recent systematic review and meta-analysis concluded that a relatively high prevalence of sleep problems emerged during the COVID-19 pandemic and females were disproportionately affected (57). In Bangladesh, females mostly engage in taking care of family members and maintain household work. Lockdown-related stressors including taking care of children and elderly family members amidst the pandemic could increase the likelihood of females developing poor sleep quality (58). In addition, there is a substantial existing literature showing that females were more prone to the poor sleep quality than males (59–62). In contrast, a few pieces of Bangladeshi research reported no gender differences in sleep quality among university students during the pre-COVID-19 periods (42, 44). This may be due to the differences on the study populations and another reason would be due to the impact of COVID-19. However, this association warrants future studies to verify this finding.

In this study, respondents with self-reported moderate/poor health status had higher odds of poor sleep quality than those who reported good health status, consistent with a previous Bangladeshi study (43) and global studies (63–65). This finding also agrees with a Chinese study conducted during the COVID-19 pandemic using similar methods (66). Patients with underlying health conditions such as chronic respiratory diseases, renal problems, and diabetes appear to be at a greater risk of morbidity or mortality from COVID-19 (66–68). So, poor health status may lead to poor sleep quality.

The present findings demonstrated decreased household income due to the impact of COVID-19 anticipating poor sleep quality, which is consistent with the COVID-19 study (14) and also with the pre-COVID-19 study (69). Previous study also indicated that increased sleep disturbances (reduced sleep duration and poor sleep quality) and the financial crisis among Greek railway workers were associated (70). A longitudinal analysis conducted among UK adults reported that worry about loss of work or decreased household income was associated with poorer sleep (71). One possible explanation could be that being jobless or having a decreased income may impact sleep only after being rejected repeatedly during the job search or when a lower income begins to have an impact on living standards (71–73).

In this study, respondents with indirect contact with COVID-19-infected persons and with fear of infection were more likely to have poorer sleep quality. It was notable that indirect contact with patients with COVID-19 had significant impacts on sleep quality compared to direct contact. Direct contact with patients with COVID-19 was insignificant in the regression model, which may be responsible for the lower percentages of participants (14.9%) with direct contact in the present samples. These findings warrant additional studies. A prior study conducted with Italian general people during the COVID-19 pandemic reported that those with uncertainty regarding possible COVID-19 infection and greater fear of contact with COVID-19-infected persons had an increased risk of developing poor sleep quality (24). This may be due to mental health concerns. A recent Bangladeshi study showed that contact (direct or indirect) with infected individuals with COVID-19 and fear of infection were significantly correlated with depression, anxiety, and stress (16). This study also found that individuals with COVID-19-induced anxiety had higher chances of poor sleep quality. Sleep hygiene and mental well-being depend positively on each other and good sleep quality can predict positive mental well-being (74, 75). Poor quality of sleep is related to poor mental health conditions (e.g., anxiety, depression, and stress) (76). In addition, a recent scoping review suggests that there is a high prevalence of commonly diagnosed psychiatric disorders such as anxiety and depression in people with obstructive sleep apnea (77).

To note, residence was statistically significant in the model that adjusted for background variables, while having infected family members was statistically significant in the model adjusted for COVID-19-related variables. Consequently, neither (either residence or having infected family members) were regressed in the model adjusted for both the background and COVID-19-related variables.

### Public Health Implication

According to the relatively high prevalence estimate of poor sleep quality found among the general population in Bangladesh during COVID-19, it seems that additional measures are required to protect this vulnerable group. The findings may draw the attention of healthcare authorities to take initiatives for improving the sleep quality of general people. Awareness programs can be initiated through television and social media to minimize COVID-19-related fear and anxiety. Moreover, the findings would contribute to baseline information in the future for longitudinal studies or other pieces of research, including interventional studies.

### Limitations

There are some drawbacks to this study. First, this study was of cross-sectional nature that could not establish causal inferences. Second, this study used an online survey method considering spatial distancing and lockdown, so the cohort represents sampling biases by being conducted online, thereby restricting itself to those with internet access and, thus, unlikely to represent an accurate representation of the entire population of Bangladesh. Moreover, compared to face-to-face interviews, self-reporting has limitations including multiple biases (e.g., social desirability, memory recall, etc.). Although this study recruited an adequate sample by using a convenience sampling technique, it cannot be considered as nationally representative given the higher proportion of higher education, urban residency, and the low average mean age of the sample. Finally, since there was no pre-COVID-19 evidence, it cannot be argued that the elevated prevalence estimate was solely due to the COVID-19 pandemic.

## Conclusion

This study provides some baseline information concerning sleep quality among Bangladeshi residents during the COVID-19 pandemic. The findings reflected a higher prevalence estimate of poor sleep quality amid this pandemic involving those who reported female gender, moderate/poor health status, contact with COVID-19-infected persons, decreased household income due to the COVID-19 pandemic, fear of infection, and COVID-19-induced anxiety. The findings suggest an immediate intervention for this vulnerable group to improve their sleep quality during the COVID-19 pandemic. These associated factors of poor sleep quality should be addressed by the respective healthcare authorities in Bangladesh to take appropriate interventions. Online counseling, awareness, and motivation need to be built in this respect.

## Data Availability Statement

The raw data supporting the conclusions of this article will be made available by the authors, without undue reservation.

## Ethics Statement

The study protocol was reviewed and approved by the Institutional Review Board of Sheik Sayera Khatun Medical College, Gopalganj, Bangladesh (SSKMC/EC/2020/810).

## Author Contributions

MI contributed to the conceptualization, investigation, methodology, data curation, formal analysis, writing—original draft, writing—review and editing, and validation. MR contributed to the conceptualization, investigation, methodology, writing—original draft, writing—review and editing, and validation. AZ and MB contributed to the investigation, data curation, writing—original draft, and validation. MK contributed to the supervision, writing—review and editing, and Validation. LH and MS contributed to the writing—review and editing and validation. All authors contributed to the article and approved the submitted version.

## Conflict of Interest

The authors declare that the research was conducted in the absence of any commercial or financial relationships that could be construed as a potential conflict of interest.

## Publisher's Note

All claims expressed in this article are solely those of the authors and do not necessarily represent those of their affiliated organizations, or those of the publisher, the editors and the reviewers. Any product that may be evaluated in this article, or claim that may be made by its manufacturer, is not guaranteed or endorsed by the publisher.

## References

[1] Cucinotta D Vanelli M. WHO declares COVID-19 a pandemic. Acta bio-medica Atenei Parm. (2020) 91:157–60. 10.23750/abm.v91i1.9397 32191675 PMC7569573

[2] World Health Organization. Coronavirus Disease (COVID-19) Pandemic. (2020). Available online at: https://www.who.int/emergencies/diseases/novel-coronavirus-2019 (accessed July 16, 2021).

[3] Star TD. Coronavirus Outbreak: Govt Orders Closure of Public, Private Offices From March 26 to April 4. (2020). Available online at: https://www.thedailystar.net/coronavirus-deadly-new-threat/news/govt-offices-closed-march-26-april-4-cabinet-secretary-1884730 (accessed July 16, 2021).

[4] Ferdous MZ Islam MS Sikder MT Mosaddek ASM Zegarra-Valdivia JA Gozal D. Knowledge, attitude, and practice regarding COVID-19 outbreak in Bangladesh: An online-based cross-sectional study. PLoS ONE. (2020) 15:e0239254. 10.1371/journal.pone.0239254 33035219 PMC7546509

[5] Institute of Epidemiology Disease Control and Research. Covid-19 Status for Bangladesh. (2021). Available online at: http://old.iedcr.gov.bd/ (accessed July 16, 2021).

[6] Star TD. Coronavirus Outbreak: Shutdown Won't be Extended After May 30. (2020). Available online at: https://www.thedailystar.net/coronavirus-outbreak-shutdown-wont-be-extended-after-may-30-1905826 (accessed July 16, 2021).

[7] Islam MS Emran GI Rahman E Banik R Sikder T Smith L. Knowledge, attitudes and practices associated with the COVID-19 among slum dwellers resided in Dhaka City: a Bangladeshi interview-based survey. J Public Health. (2021) 43:13–25. 10.1093/pubmed/fdaa182 33057666 PMC7665690

[8] Dhaka Tribune. Restriction on Public Movement Extended Till August 31. (2020). Available online at: https://www.dhakatribune.com/health/coronavirus/2020/08/03/restriction-on-public-movement-extended-till-august-31 (accessed July 16, 2021).

[9] Brooks SK Webster RK Smith LE Woodland L Wessely S Greenberg N. The psychological impact of quarantine and how to reduce it: rapid review of the evidence. Lancet. (2020) 395:912–20. 10.1016/S0140-6736(20)30460-8 32112714 PMC7158942

[10] Islam MS Potenza MN van Os J. Posttraumatic stress disorder during the COVID-19 pandemic: upcoming challenges in Bangladesh and preventive strategies. Int J Soc Psychiatry. (2020) 67:205–6. 10.1177/0020764020954469 32873127

[11] Tasnim R Sujan MSH Islam MS Ritu AH Siddique MA Toma TY. Prevalence and correlates of anxiety and depression in frontline healthcare workers treating people with COVID-19 in Bangladesh. BMC Psychiatry. (2021) 21:271. 10.1186/s12888-021-03243-w 34034679 PMC8146174

[12] Islam MS Sujan MSH Tasnim R Sikder MT Potenza MN van Os J. Psychological responses during the COVID-19 outbreak among university students in Bangladesh. PLoS ONE. (2020) 15:e0245083. 10.1371/journal.pone.0245083 33382862 PMC7775049

[13] Banerjee D. The COVID-19 outbreak: crucial role the psychiatrists can play. Asian J Psychiatr. (2020) 50:102014. 10.1016/j.ajp.2020.102014 32240958 PMC7270773

[14] Saadeh H Saadeh M Almobaideen W Al Refaei A Shewaikani N Al Fayez RQ. Effect of COVID-19 quarantine on the sleep quality and the depressive symptom levels of University Students in Jordan during the Spring of 2020. Front Psychiatry. (2021) 12:131. 10.3389/fpsyt.2021.605676 33664681 PMC7920987

[15] Islam MS Ferdous MZ Potenza MN. Panic and generalized anxiety during the COVID-19 pandemic among Bangladeshi people: an online pilot survey early in the outbreak. J Affect Disord. (2020) 276:30–7. 10.1016/j.jad.2020.06.049 32697713 PMC7362838

[16] Zubayer AA Rahman ME Islam MB Babu SZD Rahman QM Bhuiyan MRAM. Psychological states of Bangladeshi people four months after the COVID-19 pandemic: an online survey. Heliyon. (2020) 6:e05057. 10.1016/j.heliyon.2020.e05057 33015396 PMC7521899

[17] Besedovsky L Lange T Born J. Sleep and immune function. Pflugers Arch Eur J Physiol. (2012) 463:121–37. 10.1007/s00424-011-1044-0 22071480 PMC3256323

[18] Chair SY Wang Q Cheng HY Lo SWS Li XM Wong EML Sit JWH. Relationship between sleep quality and cardiovascular disease risk in Chinese post-menopausal women. BMC Womens Health. (2017) 17:1–7. 10.1186/s12905-017-0436-5 28893224 PMC5594540

[19] Léger D Bayon V. Societal costs of insomnia. Sleep Med Rev. (2010) 14:379–89. 10.1016/j.smrv.2010.01.003 20359916

[20] Cappuccio FP D'Elia L Strazzullo P Miller MA. Sleep duration and all-cause mortality: a systematic review and meta-analysis of prospective studies. Sleep. (2010) 33:585–92. 10.1093/sleep/33.5.585 20469800 PMC2864873

[21] Chattu VK Manzar MD Kumary S Burman D Spence DW Pandi-Perumal SR. The global problem of insufficient sleep and its serious public health implications. Healthcare. (2018) 7:1. 10.3390/healthcare7010001 30577441 PMC6473877

[22] Jahrami H BaHammam AS Bragazzi NL Saif Z Faris M Vitiello MV. Sleep problems during the COVID-19 pandemic by population: a systematic review and meta-analysis. J Clin Sleep Med. (2021) 17:299–313. 10.5664/jcsm.8930 33108269 PMC7853219

[23] Huang Y Zhao N. Generalized anxiety disorder, depressive symptoms and sleep quality during COVID-19 outbreak in China: a web-based cross-sectional survey. Psychiatry Res. (2020) 288:112954. 10.1016/j.psychres.2020.112954 32325383 PMC7152913

[24] Casagrande M Favieri F Tambelli R Forte G. The enemy who sealed the world: effects quarantine due to the COVID-19 on sleep quality, anxiety, and psychological distress in the Italian population. Sleep Med. (2020) 75:12–20. 10.1016/j.sleep.2020.05.011 32853913 PMC7215153

[25] Marelli S Castelnuovo A Somma A Castronovo V Mombelli S Bottoni D. Impact of COVID-19 lockdown on sleep quality in university students and administration staff. J Neurol. (2020) 268:8–15. 10.1007/s00415-020-10056-6 32654065 PMC7353829

[26] Jahrami H BaHammam AS AlGahtani H Ebrahim A Faris MAI AlEid K. The examination of sleep quality for frontline healthcare workers during the outbreak of COVID-19. Sleep Breath. (2020) 25:503–11. 10.1007/s11325-020-02135-9 32592021 PMC7319604

[27] Kumar M Kumar P. Impact of pandemic on mental health in lower- and middle-income countries (LMICs). Glob Ment Heal. (2020) 7:e35. 10.1017/gmh.2020.28 34191999 PMC7750653

[28] Josephson A Kilic T Michler JD. Socioeconomic impacts of COVID-19 in low-income countries. Nat Hum Behav. (2021) 5:557–65. 10.1038/s41562-021-01096-7 33785897

[29] Pereira M Oliveira AM. Poverty and food insecurity may increase as the threat of COVID-19 spreads. Public Health Nutr. (2020) 23:3236–40. 10.1017/S1368980020003493 32895072 PMC7520649

[30] Chackalackal DJ Al-Aghbari AA Jang SY Ramirez TR Vincent J Joshi A. The Covid-19 pandemic in low- and middle-income countries, who carries the burden? Review of mass media and publications from six countries. Pathog Glob Health. (2021) 115:178–87. 10.1080/20477724.2021.1878446 33657984 PMC8079077

[31] Banna MH Al Sayeed A Kundu S Christopher E Hasan MT Begum MR. The impact of the COVID-19 pandemic on the mental health of the adult population in Bangladesh: a nationwide cross-sectional study. Int J Environ Health Res. (2020) 2020:1–12. 10.1080/09603123.2020.1802409 32741205

[32] Islam MS Rahman ME Banik R Emran MGI Saiara N Hossain S. Financial and mental health concerns of impoverished urban-dwelling Bangladeshi people during COVID-19. Front Psychol. (2021) 12:663687. 10.3389/fpsyg.2021.663687 34421719 PMC8377359

[33] Sujan MSH Tasnim R Islam MS Ferdous MZ Apu MAR Musfique MM. COVID-19-specific diabetes worries amongst diabetic patients: the role of social support and other co-variates. Prim Care Diabetes. (2021) 15:778–85. 10.1016/j.pcd.2021.06.009 34210639 PMC8226038

[34] Hossain MM Hsan K Islam MS Nath SK. Psychological states of Bangladeshi people and associated factors during the outbreak of COVID-19: a cross-sectional survey. Emerg Trends Drugs. (2021) 1:100012. 10.1016/j.etdah.2021.100012 PMC8674962 34931182

[35] Islam MS Sujan MSH Tasnim R Mohona RA Ferdous MZ Kamruzzaman S. Problematic smartphone and social media use among Bangladeshi college and university students amid COVID-19: the role of psychological wellbeing and pandemic related factors. Front psychiatry. (2021) 12:647386. 10.3389/fpsyt.2021.647386 33935834 PMC8085355

[36] Tasnim R Islam MS Sujan MSH Sikder MT Potenza MN. Suicidal ideation among Bangladeshi university students early during the COVID-19 pandemic: Prevalence estimates and correlates. Child Youth Serv Rev. (2020) 119:105703. 10.1016/j.childyouth.2020.105703 33204046 PMC7654299

[37] Islam MS Ferdous MZ Islam US Mosaddek ASM Potenza MN Pardhan S. Treatment, persistent symptoms, and depression in people infected with COVID-19 in Bangladesh. Int J Environ Res Public Health. (2021) 18:1453. 10.3390/ijerph18041453 33562427 PMC7914967

[38] Islam MS Sujan MSH Tasnim R Ferdous MZ Masud JHB Kundu S. Problematic internet use among young and adult population in Bangladesh: Correlates with lifestyle and online activities during the COVID-19 pandemic. Addict Behav Reports. (2020) 12:100311. 10.1016/j.abrep.2020.100311 33364319 PMC7752719

[39] Safa F Anjum A Hossain S Trisa TI Alam SF Abdur Rafi M. Immediate psychological responses during the initial period of the COVID-19 pandemic among Bangladeshi medical students. Child Youth Serv Rev. (2021) 122:105912. 10.1016/j.childyouth.2020.105912 33390637 PMC7769705

[40] Mondal H Mondal S Baidya C. Comparison of perceived sleep quality among urban and rural adult population by Bengali Pittsburgh Sleep Quality Index. Adv Hum Biol. (2018) 8:36–40. 10.4103/AIHB.AIHB_44_17

[41] Ahmed MS Khan S Hsan K Sen LC Yunus FM Griffiths MD. Factors affecting sleep quality among the university students in Bangladesh: a cross-sectional structured interview study. Sleep Vigil. (2020) 4:177–84. 10.1007/s41782-020-00106-4

[42] Jahan MS Hossain SR Sayeed UB Wahab A Rahman T Hossain A. Association between internet addiction and sleep quality among students : A cross-sectional study in Bangladesh. Sleep Biol Rhythms. (2019) 17:323–9. 10.1007/s41105-019-00219-y

[43] Ahmed MS Sen L Griffiths M. Association between self-rated health and quality of life with sleep quality among Bangladeshi university students. Soc Heal Behav. (2020) 3:35–7. 10.4103/SHB.SHB_15_20

[44] Islam MZ Hsan K Islam MS Gozal D Hossain MM. Assessment of sleep quality and its association with problematic internet use among university students: a crosssectional investigation in Bangladesh. Sleep Sci. (2020). 10.5935/1984-0063.20200069. [Epub ahead of print]. PMC8663732 34917268

[45] Ara T Rahman MM Hossain MA Ahmed A. Identifying the associated risk factors of sleep disturbance during the COVID-19 lockdown in Bangladesh: a web-based survey. Front Psychiatry. (2020) 11:1–11. 10.3389/fpsyt.2020.580268 33093839 PMC7527420

[46] Ahammed B Jahan N Seddeque A Hossain MT Shovo T-E-A Khan B. Exploring the association between mental health and subjective sleep quality during the COVID-19 pandemic among Bangladeshi university students. Heliyon. (2021) 7:e07082. 10.1016/j.heliyon.2021.e07082 34095577 PMC8165399

[47] Hasan M Maliha Z Rahman A Mamun MA. Insomnia in Bangladeshi young adults during the COVID-19 pandemic: the role of behavioral factors, COVID-19 risk and fear, and mental health issues. Sleep Vigil. (2021). 10.1007/s41782-021-00161-5. [Epub ahead of print]. 34423233 PMC8366484

[48] Das R Hasan MR Daria S Islam MR. Impact of COVID-19 pandemic on mental health among general Bangladeshi population: a cross-sectional study. BMJ Open. (2021) 11:e045727. 10.1136/bmjopen-2020-045727 33837107 PMC8042595

[49] Rahman ME Moonajilin MS Bishwas MS Banik R Pinky GN Alin SI. Awareness, knowledge about human papillomavirus and attitude towards its vaccine among university students: A Bangladeshi pilot study. Asian J Heal Sci. (2019) 5:11. 10.15419/ajhs.v5i2.458

[50] Hamza MS Badary OA Elmazar MM. Cross-sectional study on awareness and knowledge of COVID-19 among senior pharmacy students. J Community Health. (2020) 46:139–46. 10.1007/s10900-020-00859-z 32542552 PMC7295146

[51] Rahman ME Islam MS Bishwas MS Moonajilin MS Gozal D. Physical inactivity and sedentary behaviors in the Bangladeshi population during the COVID-19 pandemic: an online cross-sectional survey. Heliyon. (2020) 6:e05392. 10.1016/j.heliyon.2020.e05392 33163666 PMC7598079

[52] Rahman ME Islam MS Mamun MA Moonajilin MS Yi S. Prevalence and factors associated with suicidal ideation among university students in Bangladesh. Arch Suicide Res. (2020). 10.1080/13811118.2020.1833800. [Epub ahead of print]. 33073746

[53] Lee SA. Coronavirus anxiety scale: a brief mental health screener for COVID-19 related anxiety. Death Stud. (2020) 44:393–401. 10.1080/07481187.2020.1748481 32299304

[54] Ahmed O Faisal RA Jobe MC Sharker T Lee SA. Adaptation of the Bangla version of the COVID-19 anxiety scale. Int J Ment Health Addict. (2020). 10.1007/s11469-020-00357-2. [Epub ahead of print]. 32837436 PMC7320841

[55] Buysse DJ Reynolds CF III Monk TH Berman SR Kupfer DJ. The Pittsburgh Sleep Quality Index: a new instrument for psychiatric practice and research. Psychiatry Res. (1989) 28:193–213. 2748771 10.1016/0165-1781(89)90047-4

[56] Uri M Shir G Shoham C-H Joel R Miguel MC Hagit H. Escalation of sleep disturbances amid the COVID-19 pandemic: a cross-sectional international study. J Clin Sleep Med. (2020) 17:45–53. 10.5664/jcsm.8800 32900428 PMC7849644

[57] Alimoradi Z Gozal D Tsang HWH Lin C-Y Broström A Ohayon MM. Gender-specific estimates of sleep problems during the COVID-19 pandemic: systematic review and meta-analysis. J Sleep Res. (2021). 10.1111/jsr.13432. [Epub ahead of print]. 34245055 PMC8420603

[58] Alfonsi V Gorgoni M Scarpelli S Zivi P Sdoia S Mari E. COVID-19 lockdown and poor sleep quality: Not the whole story. J Sleep Res. (2021) 30:e13368. 10.1111/jsr.13368 33955081 PMC8236908

[59] Quan SA Li YC Li WJ Li Y Jeong JY Kim DH. Gender differences in sleep disturbance among elderly Koreans: Hallym aging study. J Korean Med Sci. (2016) 31:1689–95. 10.3346/jkms.2016.31.11.1689 27709844 PMC5056198

[60] Wong WS Fielding R. Prevalence of insomnia among Chinese adults in Hong Kong: a population-based study. J Sleep Res. (2011) 20:117–26. 10.1111/j.1365-2869.2010.00822.x 20408932

[61] Manzar MD Zannat W Kaur M Hussain ME. Sleep in university students across years of university education and gender influences. Int J Adolesc Med Health. (2015) 27:341–8. 10.1515/ijamh-2014-0037 25470605

[62] Becker SP Jarrett MA Luebbe AM Garner AA Burns GL Kofler MJ. Sleep in a large, multi-university sample of college students: sleep problem prevalence, sex differences, and mental health correlates. Sleep Heal. (2018) 4:174–81. 10.1016/j.sleh.2018.01.001 29555131 PMC5863586

[63] Štefan L Sporiš G Krističević T. The associations between sleep duration and sleep quality with self-rated health in young adults: a population-based study. Int J Adolesc Med Health. (2018) 32:7. 10.1515/ijamh-2018-0007 30352026

[64] Steptoe A Peacey V Wardle J. Sleep duration and health in young adults. Arch Intern Med. (2006) 166:1689–92. 10.1001/archinte.166.16.1689 16983045

[65] Benyamini Y. Why does self-rated health predict mortality? An update on current knowledge and a research agenda for psychologists. Psychol Health. (2011) 26:1407–13. 10.1080/08870446.2011.621703 22111660

[66] Lin Y Liu S Li S Zuo H Zhang B. Relationships between the changes in sleep patterns and sleep quality among Chinese people during the 2019 coronavirus disease outbreak. Sleep Med. (2021). 10.1016/j.sleep.2021.01.021. [Epub ahead of print]. 33541805 PMC7813507

[67] Guan W-J Ni Z-Y Hu Y Liang W-H Ou C-Q He J-X. Clinical characteristics of coronavirus disease 2019 in China. N Engl J Med. (2020) 382:1708–20. 10.1056/NEJMoa2002032 32109013 PMC7092819

[68] Team CDCC-19 R. Preliminary estimates of the prevalence of selected underlying health conditions among patients with coronavirus disease 2019 - United States, February 12-March 28, 2020. Morb Mortal Wkly Rep. (2020) 69:382–6. 10.15585/mmwr.mm6913e2 32240123 PMC7119513

[69] Antillón M Lauderdale D Mullahy J. Sleep behavior and unemployment conditions. Econ Hum Biol. (2014) 14:22–32. 10.1016/j.ehb.2014.03.003 24958451 PMC4083051

[70] Nena E Steiropoulos P Papanas N Kougkas D Zarogoulidis P Constantinidis T. Greek financial crisis: from loss of money to loss of sleep? Hippokratia. (2014) 18:135–8. 25336876 PMC4201399

[71] Wright L Steptoe A Fancourt D. Are adversities and worries during the COVID-19 pandemic related to sleep quality? Longitudinal analyses of 46,000 UK adults. PLoS ONE. (2021) 16:e0248919. 10.1371/journal.pone.0248919 33765097 PMC7993810

[72] Wanberg C Basbug G Van Hooft EAJ Samtani A. Navigating the black hole: explicating layers of job search context and adaptational responses. Pers Psychol. (2012) 65:887–926. 10.1111/peps.12005

[73] Wanberg CR Zhu J Kanfer R Zhang Z. After the pink slip: applying dynamic motivation frameworks to the job search experience. Acad Manag J. (2012) 55:261–84. 10.5465/amj.2010.0157

[74] Magnavita N Garbarino S. Sleep, health and wellness at work: a scoping review. Int J Environ Res Public Health. (2017) 14:1347. 10.3390/ijerph14111347 29113118 PMC5707986

[75] Kawada T. Feeling refreshed by sleep can predict psychological wellbeing assessed using the general health questionnaire in male workers: a 3-year follow-up study. Psychiatry Investig. (2012) 9:418–21. 10.4306/pi.2012.9.4.418 23251209 PMC3521121

[76] Bavafa A Khazaie H Khaledi-Paveh B Rezaie L. The relationship of severity of symptoms of depression, anxiety, and stress with sleep quality in earthquake survivors in Kermanshah. J Inj Violence Res. (2019) 11:225–32. 10.5249/jivr.v11i2.1203 31263090 PMC6646827

[77] Gupta MA Simpson FC. Obstructive sleep apnea and psychiatric disorders: a systematic review. J Clin Sleep Med. (2015) 11:165–75. 10.5664/jcsm.4466 25406268 PMC4298774

